# Intestinal microenvironment dynamics and Sepsis-associated encephalopathy pathophysiology: insights from multi-omics profiling

**DOI:** 10.3389/fneur.2025.1724644

**Published:** 2026-01-26

**Authors:** Zhou Xing Zhang, Wen Bo Xu, Fu Li Gu, Yue Chen Zhang, Wei Hu, Shao Song Xi

**Affiliations:** 1 Affiliated Hangzhou First People’s Hospital, School of Medicine, Westlake University, Hangzhou, Zhejiang, China; 2 The Fourth School of Clinical Medicine, Hangzhou First People’s Hospital, Zhejiang Chinese Medical University, Hangzhou, Zhejiang, China; 3 Department of Critical Care Medicine, Sir Run Run Shaw Hospital, Zhejiang University School of Medicine, Hangzhou, Zhejiang, China; 4 Department of Intensive Care Unit, Hangzhou Hospital of Traditional Chinese Medicine, Hangzhou TCM Hospital Affiliated to Zhejiang Chinese Medical University, Hangzhou, Zhejiang, China

**Keywords:** biomarker, gut microbiota, gut–brain axis, microRNA, sepsis-associated encephalopathy

## Abstract

**Background:**

Sepsis-associated encephalopathy (SAE), a devastating complication of sepsis, lacks specific biomarkers and clear pathophysiological understanding, particularly regarding the gut-brain axis. While gut dysbiosis is implicated in SAE, the underlying mechanisms remain elusive.

**Methods:**

This study employed an integrated multiomics approach (16S rDNA and fecal miRNA sequencing) to dissect the gut microenvironment in SAE patients (*n* = 10) compared to sepsis patients without encephalopathy (SP, *n* = 20).

**Results:**

Although *α*- and *β*-diversity indices showed no significant differences, distinct compositional shifts in the gut microbiota were observed in SAE patients, characterized by increased abundance of *Neisseria, Haemophilus, Lautropia, Enterococcus, Parabacteroides*, and decreased *Fusobacterium, Phocaeicola, Bacteroides*, among others. Concurrently, 12 fecal miRNAs were differentially expressed (DE) in SAE, with 11 upregulated (e.g., miR-106a-5p, miR-181a-5p, miR-223-5p, miR-30e-3p) and 1 downregulated (miR-222-3p). Crucially, correlation network analysis revealed significant interactions between 10 DE miRNAs and 15 bacterial genera, establishing a complex gut microbiota-miRNA interplay in SAE. Machine learning (LASSO and elastic net regression) identified miR-30e-3p and miR-223-5p as the most promising combined diagnostic biomarkers, achieving an area under the curve (AUC) of 0.893. Functional exploration via ceRNA network analysis indicated miR-30e-3p targets inflammation and apoptosis-related genes (e.g., IL1B, RPS6KB1, AKT1), while miR-223-5p primarily targets immune-regulatory genes (e.g., IGF1, AR). Experimental validation confirmed significantly elevated serum IL-1β levels in SAE patients (*p* < 0.001), supporting the predicted inflammatory pathway.

**Conclusion:**

This study provides the first evidence of a fecal miRNA-gut microbiota interaction network in SAE pathogenesis, highlighting miR-30e-3p and miR-223-5p as pivotal mediators and potential diagnostic/therapeutic targets.

## 1 Introduction

Sepsis-associated encephalopathy (SAE), a diffuse cerebral dysfunction complicating sepsis, presents as a spectrum of neuropsychiatric symptoms from delirium to coma (1, 2). It portends a grim prognosis, with mortality exceeding 50% and survivors often facing significant long-term cognitive deficits (3, 4). The underpinning pathophysiology is complex, emerging evidence highlights neuroinflammation as a central mechanism in the pathogenesis of SAE, with interleukin-1β (IL-1β) identified as a key proinflammatory cytokine (5, 6). Our previous studies demonstrated that IL-1β promotes hippocampal neuron damage in SAE rats in an autophagy-dependent manner (7).

The gut-brain axis has emerged as a critical pathway in this process (8, 9). Sepsis-induced dysbiosis can compromise intestinal barrier integrity, facilitating the translocation of inflammatory mediators and microbial products into the circulation, which may ultimately disrupt blood–brain barrier function and exacerbate neuroinflammation (10). Beyond the microbiota itself, host-derived microRNAs (miRNAs) have gained recognition as key post-transcriptional regulators of gene expression in both physiological and pathological states (11). Intriguingly, a bidirectional relationship exists where the gut microbiota can influence host miRNA expression, and miRNAs can, in turn, shape the microbial community (12). Despite this, the specific role of fecal miRNAs, and their interactions with the gut microbiota in SAE, remains entirely unexplored. This constitutes a significant knowledge gap, as fecal miRNAs represent a stable, non-invasive window into the host’s intestinal mucosal response and a potential mechanism for gut-brain communication.

To address this, we employed an integrated multi-omics approach. We hypothesized that SAE patients exhibit a distinct gut microbiota composition and fecal miRNA signature, and that specific interactions between them contribute to disease pathogenesis and could serve as novel diagnostic biomarkers. We characterized the gut microbiome and fecal miRNAome in a well-stratified cohort of SAE and sepsis patients, integrated these datasets to construct a correlation network, and utilized machine learning to identify and validate a diagnostic miRNA panel while exploring its functional relevance.

## 2 Methods

### 2.1 Ethics statement

This study was approved by the Ethics Committee of Hangzhou First People’s Hospital (Ethics Approval Certificate: KY-20211105-0078-01) and was registered in the Chinese Clinical Trial Registry (Registration Number: ChiCTR2400090085).

### 2.2 Study design and population

A total of 89 patients admitted to the Department of Critical Care Medicine and Emergency Department of Hangzhou First People’s Hospital between April 2021 and October 2022 were recruited. They were assigned to the SAE group or the non-SAE group according to the Sepsis-3 diagnostic criteria (13). The detailed inclusion and exclusion criteria, which operationalize Sepsis-3 for our clinical setting, are provided in Supplementary Text S1.

### 2.3 Collection of biological samples

The perianal area was cleaned with 70% alcohol, and then a sterile swab moistened with saline was inserted into the anus 4 ~ 5 cm from the anal sphincter and gently rotated to obtain a fecal sample from the anal crypt (traces of feces were clearly visible on the swab). The sample was then inserted into a sterile airtight test tube and immediately transferred to a − 80 °C refrigerator for freezing and storing (14).

### 2.4 Analysis of species diversity, community structure, and differential microbial taxon abundance

The *α* diversity of the gut microbiota was evaluated using QIIME (version 1.9.1) by calculating the Chao1, Invsimpson, Shannon, Simpson, and richness indices. Rank-abundance and species accumulation curves were generated using R software (version 4.2.1), which was also used for the statistical analysis of the intergroup differences in αdiversity. *β* diversity was assessed on the basis of UniFrac metrics computed in QIIME (version 1.9.1), and PCoA plots were constructed using the ade4 and ggplot2 packages in R (15). LEfSe was performed with LEfSe software, applying a LDA score threshold of 3 as the default criterion for feature selection. Analysis of similarity (ANOSIM) was conducted using the vegan package in R. Differentially abundant taxa between groups were identified using t tests and were visualized in R to present the findings (16).

Other materials and methods are described in Supplementary Text S2.

### 2.5 Statistical analysis

The quantitative data were tested for normality, and normally distributed variables are presented as the means ± standard deviations. A t test was used for comparisons between two groups, and analysis of variance (ANOVA) was used for comparisons among multiple groups. Normally distributed quantitative data are presented as the median and interquartile range, and comparisons between groups were made via two or more multigroup rank-sum tests. Qualitative data are expressed as percentages, and comparisons between groups were made via the stratified chi-square test or Fisher’s exact probability method. Spearman’s rank correlation test was used for correlation analysis, and Student’s t test was used for comparisons of diversity indices. *p* < 0.05 was considered to indicate a statistically significant difference (17).

The baseline characteristics of the groups were compared using the CBCgrps package (v2.1) in R, which is designed for observational studies. Appropriate tests (e.g., *t*-tests, chi-square tests, and Fisher’s exact tests) on the basis of the data type were automatically applied (18).

## 3 Results

### 3.1 Patient characteristics and clinical parameters

**Figure 1:**
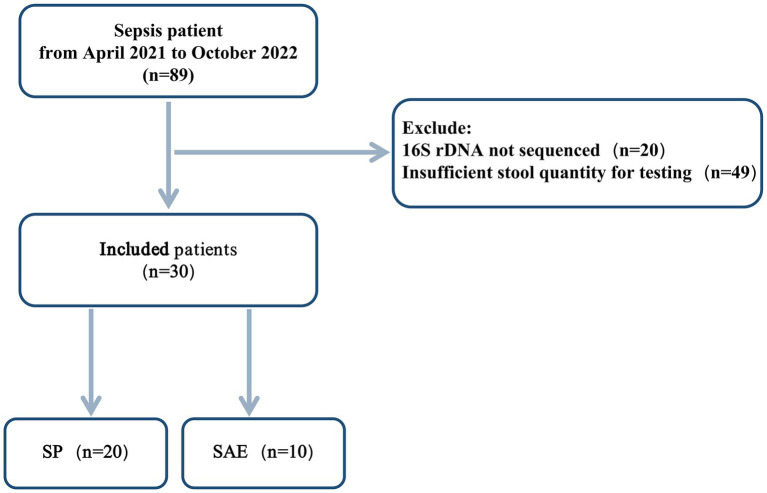
Flow chart of patient cohorts. SP: Sepsis; SAE: Sepsis-associated encephalopathy.

A total of 30 patients were included in the final analysis, comprising 20 with sepsis (SP) and 10 with sepsis-associated encephalopathy (SAE) (Figure 1). The baseline demographic and clinical characteristics were well-matched between groups, including age, sex, underlying diseases, severity scores (SOFA and APACHE II), and therapeutic interventions (*p* > 0.05, Supplementary Table S1). As expected, neurological impairment, reflected by Glasgow Coma Scale (GCS) scores, was significantly more severe in the SAE group (*p* = 0.001). Although inflammatory markers such as tumor necrosis factor-alpha (TNF-*α*), interleukin-6 (IL-6), and C-reactive protein (CRP) showed a trend toward elevation in SAE patients, these differences did not reach statistical significance (*p* > 0.05, Supplementary Table S2). However, serum lactate levels were markedly higher in the SAE group (*p* = 0.048), suggesting enhanced metabolic stress, and the PO₂/FiO₂ ratio was significantly lower (*p* = 0.044), indicating worse tissue oxygenation (Supplementary Table S2). These clinical features are consistent with recent SAE cohorts, where reduced GCS scores and higher lactate burdens have been associated with more severe encephalopathy and worse neurological outcomes despite similar baseline illness severity (19).

### 3.2 Altered gut microbiota composition in SAE

**Figure 2:**
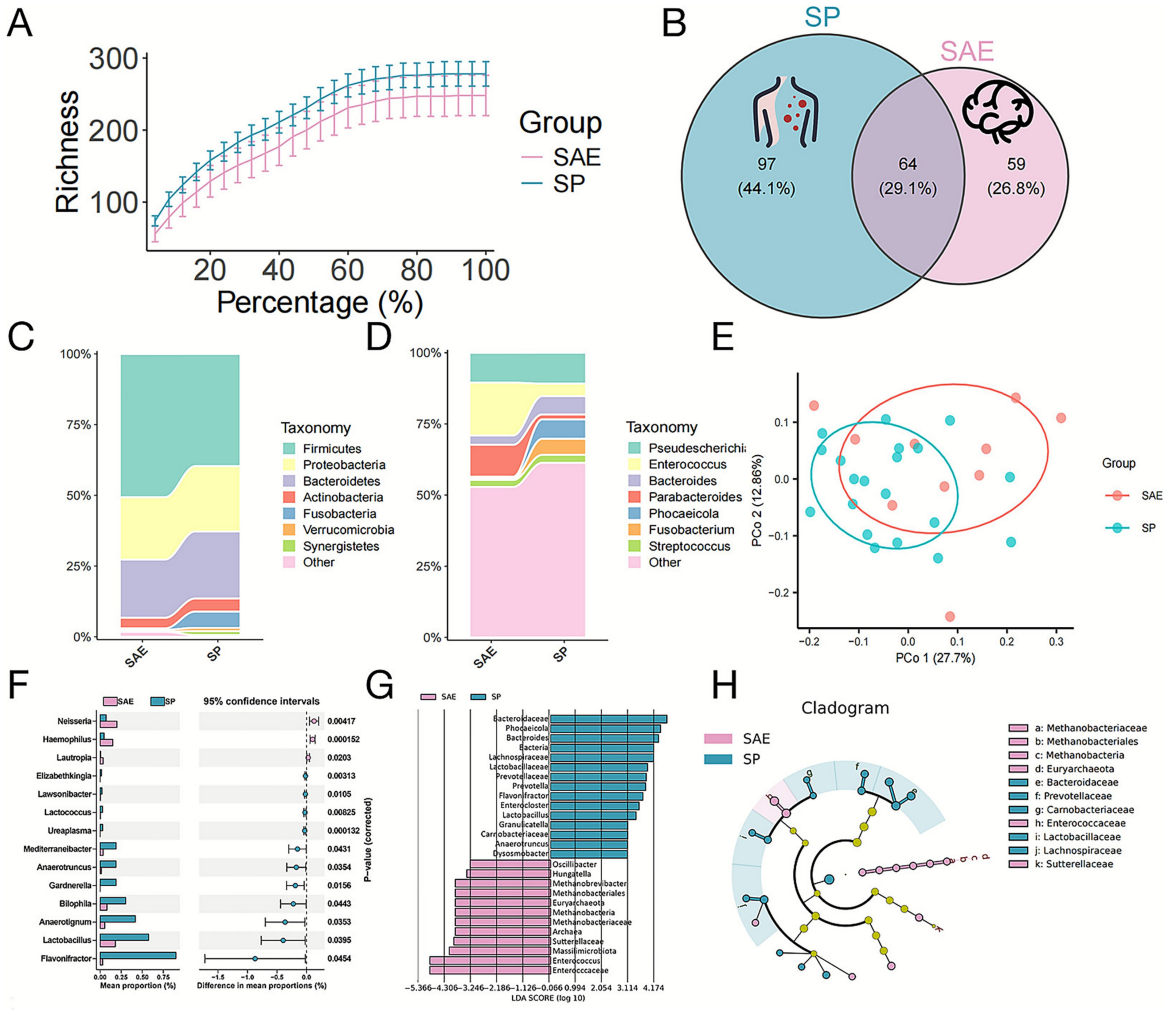
Alterations in the structure of the intestinal microbiota in patients with SP and SAE. Fecal samples from patients were collected immediately after diagnosis. **(A)** Rarefaction curves for SP and SAE samples. **(B)** Venn diagram of the ASV distribution. The overlapping parts of the circles represent the number of ASVs common to both groups/samples, and the nonoverlapping parts represent the number of ASVs specific to each group/sample. **(C)** Relative abundance of the intestinal microbiota at the phylum level. **(D)** Relative abundance of the intestinal microbiota at the genus level. **(E)** PCoA of the microbiota based on the unweighted UniFrac distance. **(F)** Bar charts illustrating the proportional representation of differentially abundant genera identified via t tests. **(G)** The LDA values for bacterial taxa exhibiting significant differences between groups (LDA > 3). The pink bars represent taxa enriched in SAE samples, whereas the blue bars represent taxa enriched in SP samples. **(H)** Taxonomic profiles obtained from LEfSe analysis of 16S rDNA sequences.

To investigate whether gut microbiota dysbiosis contributes to the pathophysiology of SAE, we performed 16S rDNA gene sequencing on fecal samples from SAE and SP patients. This approach allows for comprehensive profiling of intestinal microbial communities and has been widely applied for detecting microbial alterations in infectious diseases (20). Rarefaction curves indicated sufficient sequencing depth (Figure 2A), and a total of 220 amplicon sequence variants (ASVs) were identified, with 64 shared between groups, 97 unique to SP, and 59 unique to SAE (Figure 2B). Comparative analysis showed a significant increase in the phylum *Firmicutes* in SAE (Figure 2C), and at the genus level, *Enterococcus* and *Parabacteroides* were enriched, whereas *Fusobacterium*, *Phocaeicola*, and *Bacteroides* were reduced (Figure 2D). Principal coordinate analysis (PCoA) based on unweighted UniFrac distances showed clear separation between groups (Figure 2E), supporting distinct microbial communities. Differential abundance analysis identified *Neisseria* (*p* = 0.004), *Haemophilus* (*p* < 0.001), and *Lautropia* (*p* = 0.020) as significantly enriched in SAE, and *Flavonifractor* (*p* = 0.046), *Lactobacillus* (*p* = 0.040), and *Anaerotignum* (*p* = 0.035) as depleted (Figure 2F). Linear discriminant analysis effect size (LEfSe) analysis further confirmed enrichment of *Oscillibacter*, *Hungatella*, *Methanobrevibacter*, and *Enterococcus*, and reduction of *Phocaeicola*, *Bacteroides*, and *Lactobacillus* in SAE (Figures 2G,H). Similar patterns of sepsis-related gut dysbiosis, including enrichment of opportunistic pathobionts such as Enterococcus and loss of commensal Bacteroides and Lactobacillus, have been reported in experimental and clinical studies of SAE and sepsis, and have been linked to blood–brain barrier disruption and cognitive impairment along the gut-brain axis (21).

**Figure 3:**
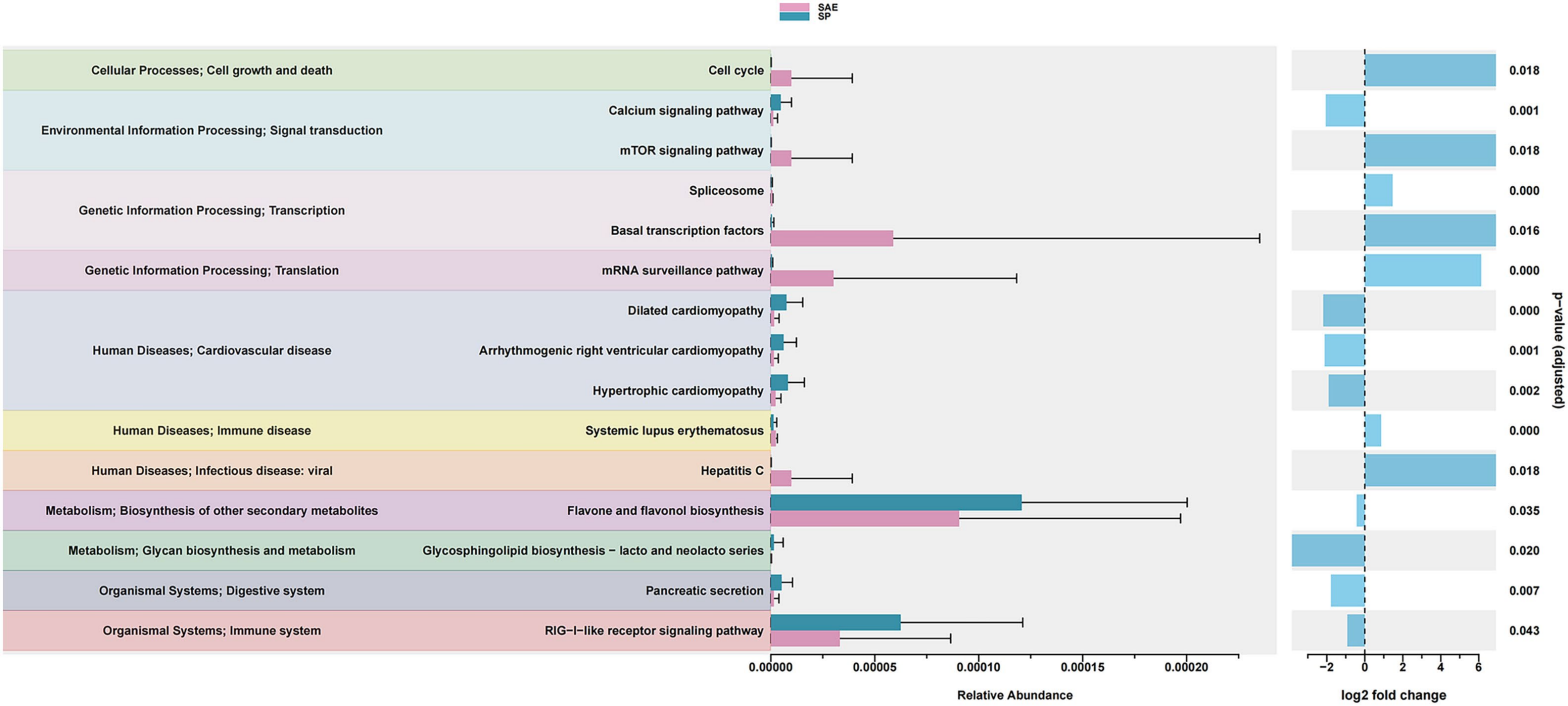
Functional predictions of microbial species associated with differentially abundant miRNAs between the SAE and SP groups. The bar charts on the left show the relative abundance of species enriched in different KEGG pathways across different functional categories, with the bars color-coded for each group (SAE in pink, SP in blue). The error bars indicate the standard deviation. The right panel shows the log2-fold change between groups for each pathway, along with adjusted *p*-values calculated using the Wilcoxon rank-sum test. Only pathways associated with significantly differentially expressed miRNAs (*p* < 0.05) are displayed. The functional categories are annotated on the basis of KEGG levels 2 and 3.

We further investigated the functional implications of the observed microbial dysbiosis by applying PICRUSt, which predicts microbial metabolic pathways based on 16S rDNA profiles. This analysis helps reveal potential biological processes influenced by gut microbiota alterations in SAE (22). Functional prediction indicated that the dysregulated microbiota was associated with pathways involving cell growth and death, signal transduction, immune regulation, and infectious disease responses (Figure 3). Specifically, the SAE group demonstrated higher abundance of species enriched in pathways such as Cell Cycle, mTOR Signaling Pathway, Basal Transcription Factors, mRNA Surveillance Pathway, Hepatitis C, Spliceosome, and Systemic Lupus Erythematosus, highlighting distinct functional and metabolic differences between the groups (Figure 3). These functionally enriched pathways are in line with recent systems-level work on the microbiota-brain axis in sepsis, which implicates microbially driven immune, cell-cycle and infection-related signaling cascades in amplifying neuroinflammation and organ dysfunction (23).

### 3.3 Differential expression of fecal miRNAs

**Figure 4:**
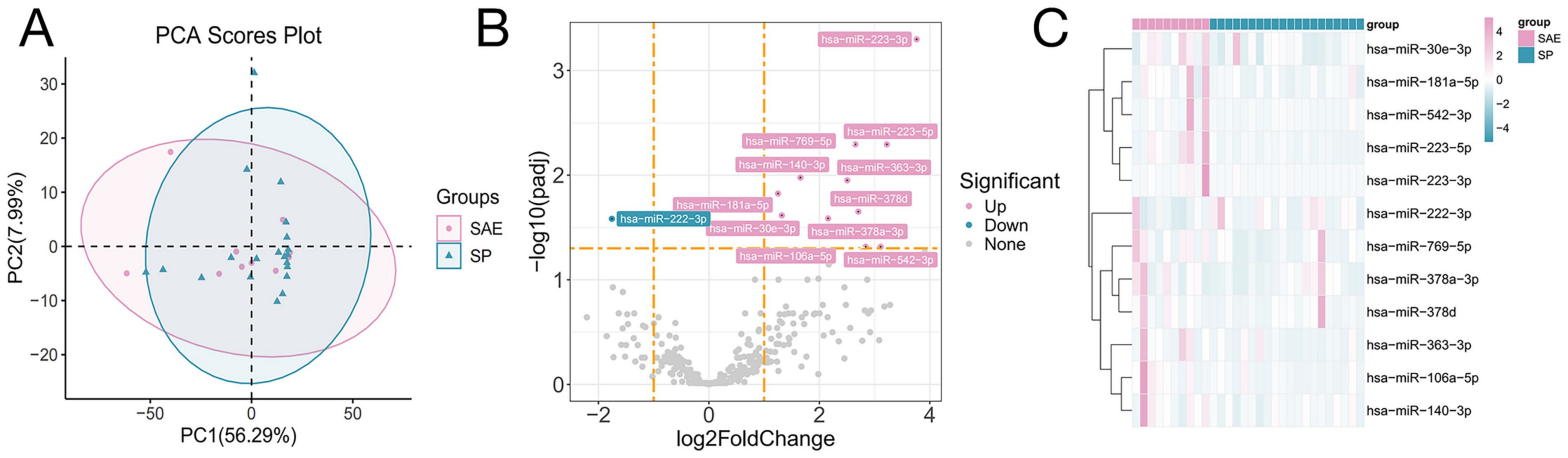
Changes in fecal miRNA expression in patients with sepsis and SAE. **(A)** PCA. **(B)** Volcano plot displaying differentially abundant miRNAs between the SAE and SP groups. The x-axis represents the log₂-fold change (SAE/SP), and the y-axis represents the −log₁₀ *p* value. **(C)** Heatmap showing significantly differentially abundant miRNAs in feces samples between the SAE and SP groups. The rows represent individual miRNAs, and the columns represent samples. The color intensity indicates normalized expression levels (Z scores), with pink denoting higher expression and teal denoting lower expression. miRNAs were selected on the basis of the log₂-fold change (SAE/SP) and statistical significance.

Small RNA sequencing identified 321 miRNAs across all samples. To explore alterations in miRNAs within the intestinal microenvironment of patients with SAE, we conducted miRNA sequencing analysis (24). A total of 321 miRNAs were identified across all the samples. Alignment of these miRNAs with the human miRNAs in the miRBase v22 database allowed us to visualize sample clustering via principal, which showed clear separation between SAE and SP groups (Figure 4A).

To elucidate the differences in the expression of miRNAs within the gut microenvironment between patients with SAE and those with SP alone, we performed differential expression analysis (DEA) of the miRNA sequencing data using the DESeq2 package (25). By applying stringent criteria of counts per million (CPM) ≥ 1, *p* < 0.05, and log2FoldChange (log2FC) ≥ 1 (26), we identified 12 significantly dysregulated miRNAs: 11 were upregulated (miR-106a-5p, miR-181a-5p, miR-223-5p, miR-223-3p, miR-140-3p, miR-30e-3p, miR-363-3p, miR-378a-3p, miR-542-3p, miR-769-5p, miR-378d) and one, miR-222-3p, was downregulated (Figures 4B,C and Supplementary Table S3). These results highlight potential miRNA signatures associated with SAE pathophysiology. Previous studies have mainly focused on circulating or brain-expressed miRNAs, such as plasma miR-370-3p and other sepsis-related miRNAs, as diagnostic and prognostic biomarkers; our data extend this work by identifying fecal miRNA signatures that may reflect local intestinal contributions to SAE pathophysiology and complement systemic markers (27).

### 3.4 Integrated microbiota–miRNA correlation network

**Figure 5:**
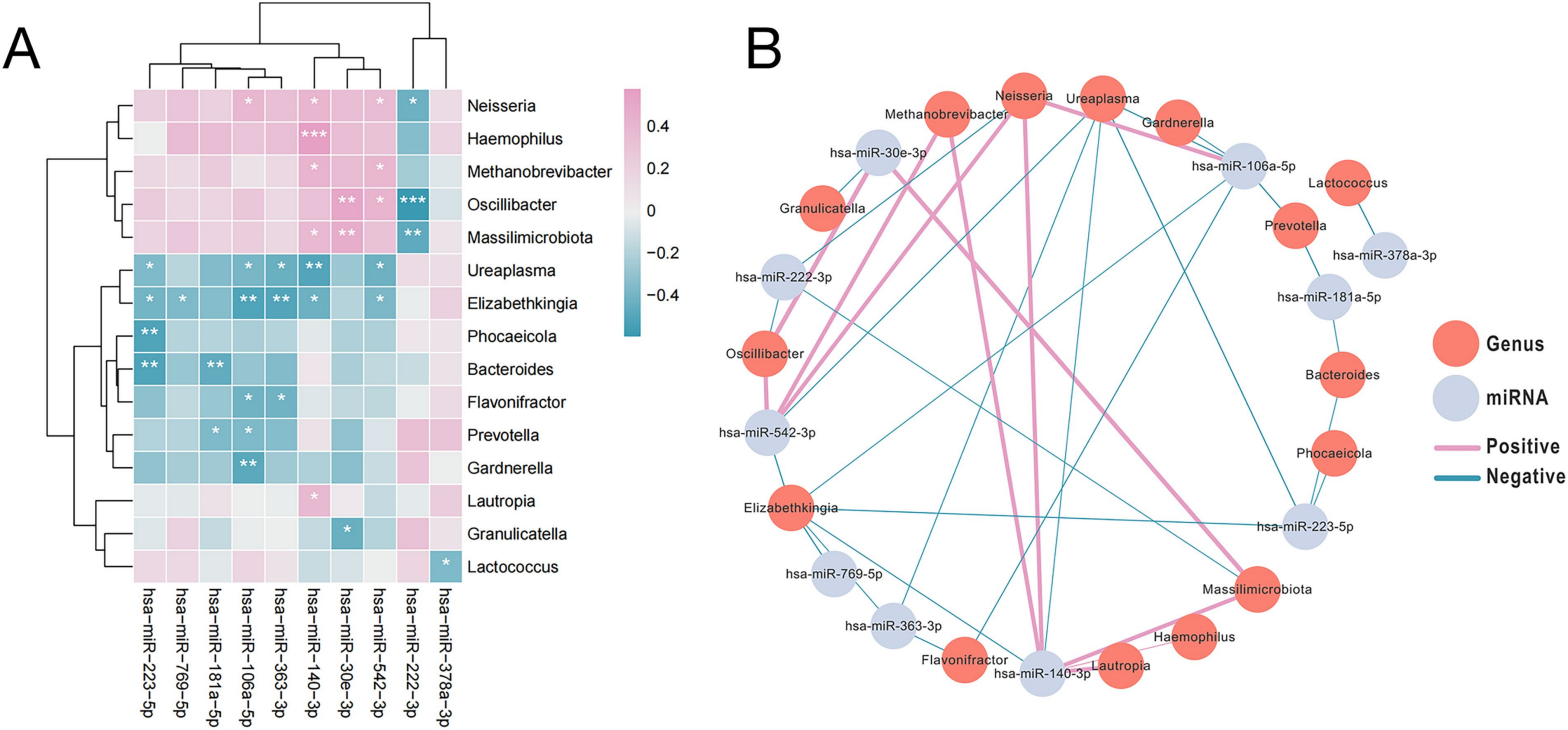
The abundance of microbial species enriched in SAE samples was significantly correlated with the expression of the most markedly differentially expression miRNAs. **(A)** Heatmap depicting the top 10 highly significantly differentially expressed miRNAs with expression levels correlated with the abundance of SAE-associated microbial species. Pink hues signify positive correlations, whereas blue tones indicate negative correlations. **(B)** The interaction diagram illustrates the 10 differentially expressed miRNAs along with their associated bacterial genera. Each vertex signifies either a miRNA or a bacterium. The edge width indicates the strength of the association, with pink edges denoting positive associations and blue edges denoting negative associations. The asterisks indicate levels of statistical significance as follows: *p < 0.05, **p < 0.01, and ***p < 0.001.

To investigate potential links between fecal miRNAs and gut microbiota in SAE, we performed correlation analysis between 10 differentially expressed miRNAs and 15 bacterial genera. Using an FDR-adjusted *p*-value < 0.05 as the significance threshold, we identified multiple significant pairwise interactions and visualized them in a co-occurrence network that highlights both positive and negative associations (Figures 5A,B and Supplementary Table S4) (28). For instance, miR-106a-5p correlated positively with *Neisseria* and negatively with *Prevotella* and *Flavonifractor*; miR-181a-5p was negatively correlated with *Prevotella* and *Bacteroides*; miR-140-3p correlated positively with *Methanobrevibacter*, *Haemophilus, Neisseria,* and negatively with *Ureaplasma* and *Elizabethkingia*. These correlation patterns are consistent with emerging evidence that fecal miRNAs can both shape and respond to gut microbial communities, forming part of a bidirectional microbiota-miRNA axis that is particularly relevant in disorders of the gut-brain axis (29).

Furthermore, the association between these SAE-associated miRNAs and genera with clinical parameters were detected by Pearson correlation analysis revealed that certain SAE-associated miRNAs and microbial genera were significantly associated with clinical parameters: *Ureaplasma* abundance was negatively associated with GCS score (rho = −0.509, *p* = 0.004), *Neisseria* abundance correlated positively with IL-2 (rho = 0.526, *p* = 0.003), and both miR-106a-5p (rho = 0.525, p = 0.003) and miR-140-3p (rho = 0.549, *p* = 0.002) expression correlated positively with lactate levels (Supplementary Table S5).

### 3.5 Machine learning-based biomarker selection

**Figure 6:**
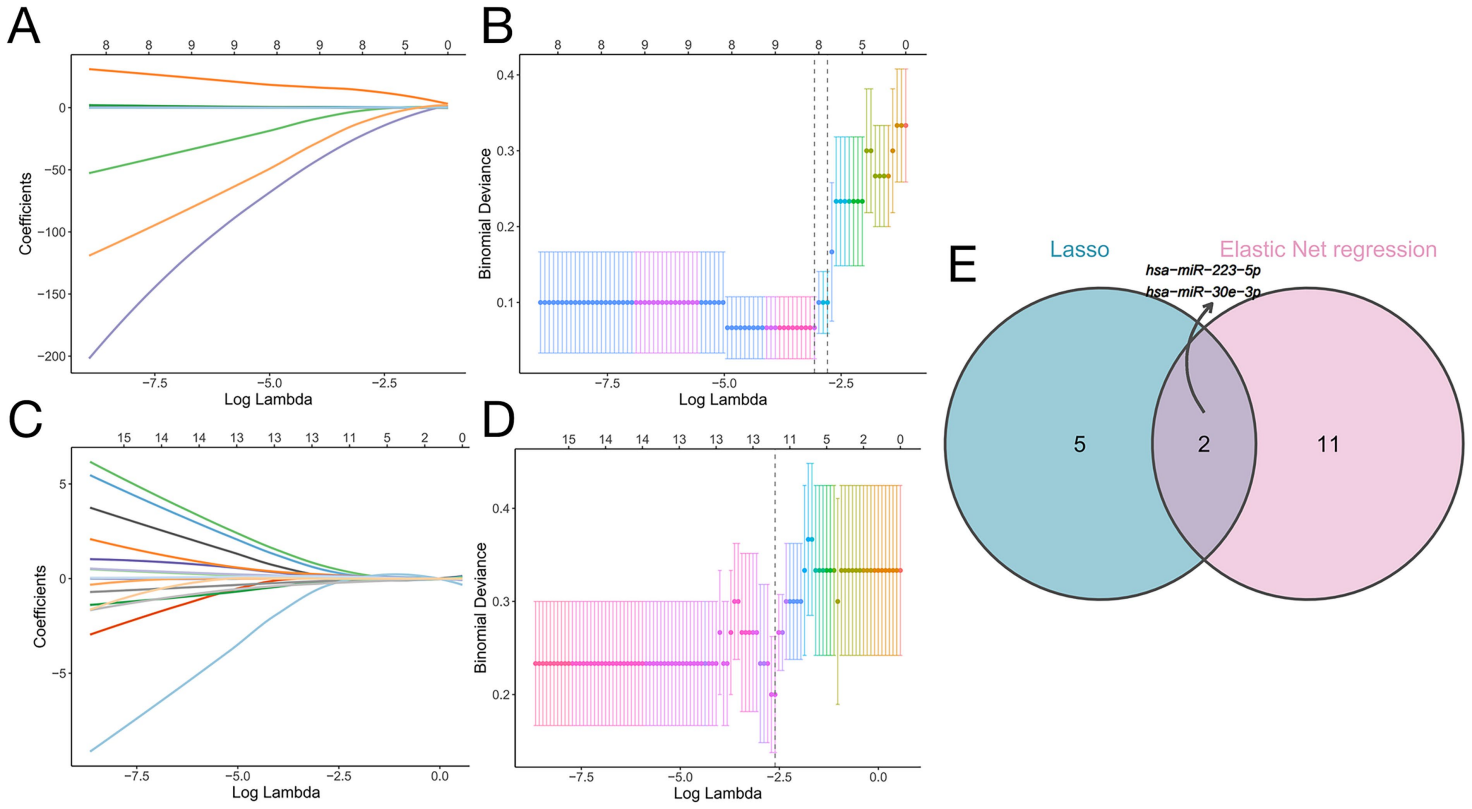
Machine learning-based identification of biomarkers distinguishing SP from SAE. **(A)** LASSO coefficient profiles of the 25 risk factors. **(B)** Binomial deviance plot for LASSO Cox regression showing the selection of seven optimal variables at the log(lambda)1se threshold (vertical dashed line). **(C)** Coefficient trajectories of the 25 candidates feature according to elastic net regression. **(D)** Binomial deviance plot of the elastic net regression results identifying 13 optimal variables, as determined by the log(lambda)1se threshold (vertical dashed line). **(E)** Venn diagram illustrating the intersection of selected features according to LASSO Cox regression and elastic net regression. There were two shared biomarkers: hsa-miR-223-5p and hsa-miR-30e-3p.

To identify optimal biomarkers distinguishing SAE from SP, we applied LASSO and elastic net regression to the 25 candidate features (10 miRNAs + 15 genera). These penalized regression methods shrink less informative coefficients toward zero, thereby performing variable selection in a high-dimensional setting (30). The coefficient trajectories of these 25 factors under varying regularization strengths are presented in Figure 6A. Using penalized regression with the log(lambda)1se criterion, we narrowed the selection to seven optimal variables (Figure 6B). Concurrently, elastic net regression was utilized, resulting in the identification of 13 potential biomarkers (Figures 6C,D). Because it is a hybrid regularization technique, elastic net regression allows for more reliable selection when the predictors are highly correlated. To pinpoint the most robust discriminative biomarkers for the two diseases, we intersected the outputs from both algorithms, ultimately identifying two highly promising candidates -hsa-miR-30e-3p and hsa-miR-223-5p - as the most robust biomarkers (Figure 6E). Our modeling strategy is in line with recent microbiome and multi-omics studies that use penalized regression (LASSO and elastic net) to derive multi-feature biomarker panels, and with best-practice recommendations for building robust microbiome-based diagnostic classifiers (30).

### 3.6 Diagnostic efficacy and functional mechanisms

**Figure 7:**
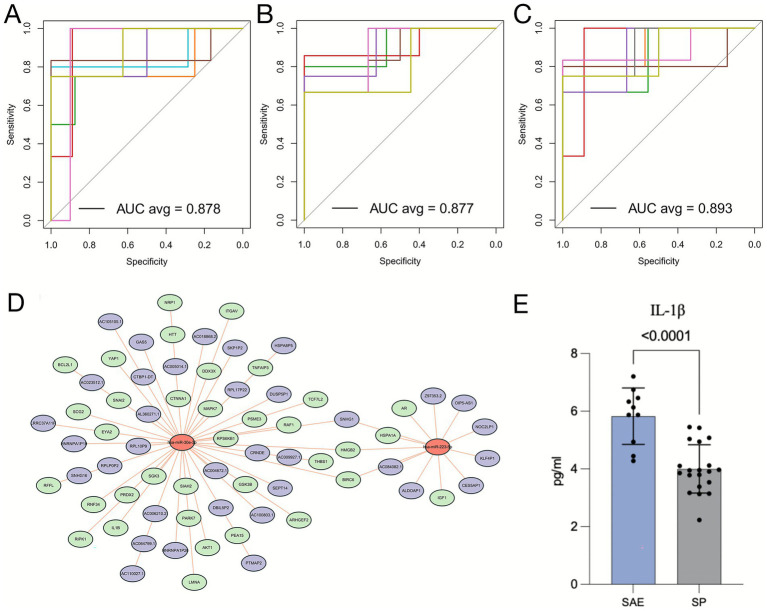
Diagnostic potential of hsa-miR-30e-3p and hsa-miR-223-5p for and mechanistic roles of these miRNAs in SAE pathogenesis. **(A-C)** ROC curves illustrating the diagnostic performance of hsa-miR-30e-3p **(A)**, hsa-miR-223-5p **(B)**, and their combination **(C)** in distinguishing SAE patients from SP patients. **(D)** ceRNA network analysis of hsa-miR-30e-3p and hsa-miR-223-5p. **(E)** Quantification of IL-1β levels in the SAE and SP groups.

The diagnostic performance of the identified biomarkers was assessed through receiver operating characteristic (ROC) analysis (31). Receiver operation characteristic analysis demonstrated high diagnostic accuracy for both miRNAs individually: miR-30e-3p (AUC = 0.878) and miR-223-5p (AUC = 0.877) (Figures 7A,B). Their combination further improved performance (AUC = 0.893) (Figure 7C).

To further investigate the potential mechanistic roles of these biomarkers in SAE, we constructed a competing endogenous RNA (ceRNA) network based on their predicted regulatory interactions. Because ceRNA analysis provides an effective framework to elucidate how miRNAs regulate downstream mRNAs through post-transcriptional inhibition and is widely used to uncover molecular mechanisms in inflammatory and neurological diseases (32), this approach enables a more comprehensive interpretation of the functional consequences of miRNA dysregulation in SAE. Functional annotation revealed that miR-30e-3p targets genes involved in inflammation (*IL-1β*) and apoptosis (*RPS6KB1*, *AKT1*), while miR-223-5p regulates immune-related genes (*IGF1*, *AR*) (Figure 7D). In support of these predictions, ELISA validation confirmed significantly elevated serum IL-1β levels in the SAE group (*p* < 0.001; Figure 7E), underscoring the relevance of inflammatory activation in SAE pathogenesis. Our ceRNA network analysis is consistent with recent sepsis studies showing that miRNA-centered ceRNA modules converge on inflammatory and immune pathways in septic organ injury, and with accumulating evidence that miR-223 in particular restrains IL-1β–driven inflammation via NLRP3- and AKT-related signaling (32).

## 4 Discussion

The findings of this comprehensive multi-omics investigation provide compelling and novel insights into the pathogenesis of SAE, establishing the gut-microbiota-fecal miRNA axis as a critical component in disease development and progression. By employing an integrated approach combining 16S rDNA sequencing and deep miRNA profiling, we have demonstrated that SAE is characterized not only by distinct gut microbial ecology but also by a specific fecal miRNA expression signature that reflects and potentially mediates the neurological complications of sepsis. More significantly, our construction of the first interaction network linking these two omics layers in SAE patients reveals a complex web of correlations that positions fecal miRNAs as key mediators of gut-brain communication in this devastating condition. The culmination of our analytical pipeline - the identification and validation of a two-miRNA panel (miR-30e-3p and miR-223-5p) with exceptional diagnostic accuracy, functionally linked to central inflammatory pathways through both bioinformatic prediction and experimental validation - represents a substantial advance in the field with immediate translational implications.

Our data reinforce and expand the growing consensus that gut dysbiosis constitutes a critical component of sepsis pathophysiology with particular relevance to neurological complications. The SAE-associated microbiota signature, characterized by enrichment in genera such as *Neisseria*, *Haemophilus*, and *Enterococcus* alongside depletion of beneficial taxa including *Lactobacillus* and *Bacteroides*, reflects a state of profound ecological disruption that extends beyond the general septic response (33, 34). This specific dysbiosis landscape, predicted through PICRUSt analysis to be functionally enriched in pro-inflammatory and immune-related pathways including mTOR signaling, likely contributes to a leaky gut phenotype that facilitates translocation of microbial products and subsequent amplification of the systemic inflammatory cascade (35, 36). Such translocation of microbial products and resultant systemic inflammation may potentiate immune effector mechanisms including neutrophil activation and NET formation, processes increasingly recognized as pivotal in sepsis-associated organ damage (37). The altered gut-microbiota, coupled with miRNA changes, therefore may represent an upstream regulatory layer of these downstream immune cascades. The statistically significant correlation between the abundance of specific bacteria and clinical markers of neurological decline (GCS score) substantially strengthens the argument for their clinical relevance and potential contributory role in driving brain dysfunction, moving beyond association toward potential causation (10).

The most groundbreaking aspect of our work lies in its successful integration of the microbiome with the host’s miRNA response, moving beyond a simple description of dysbiosis to elucidate their intricate interplay. The robust correlations we uncovered suggest a dynamic, bidirectional crosstalk that may form the basis of novel mechanistic pathways in SAE. Given that most prior sepsis studies have largely relied on peripheral blood cytokine measurements or immune cell profiling to characterize systemic inflammation and disease severity in septic patients (38), our multi-omics stool-based approach provides a non-invasive window into gut-derived regulatory signals. This strategy may help uncover previously unrecognized gut–brain communication pathways in SAE. For instance, the negative correlation between miR-181a-5p and SCFA-producing genera (*Prevotella*, *Bacteroides*) is particularly intriguing from a pathophysiological perspective (39–41). Given that SCFAs are crucial microbial metabolites known to maintain gut barrier integrity and exert potent anti-inflammatory effects on the brain through multiple mechanisms (42), their depletion coupled with the concurrent upregulation of miR-181a-5p, a miRNA previously demonstrated to aggravate neuroinflammation and cognitive deficits in neurological disorders (39), suggests a potential vicious cycle whereby microbial dysbiosis and host miRNA response mutually reinforce each other. This miRNA might not only represent a host response to microbial changes but could also actively suppress the growth of beneficial microbes, thereby exacerbating dysbiosis and neuroinflammation through a positive feedback loop. Similarly, the positive correlation between the pro-inflammatory miR-106a-5p (known to promote microglial inflammation in cerebral ischemia (43)) and *Neisseria* (a genus with documented potential for CNS invasion through paracellular pathways and infection of trigeminal Schwann cells (44, 45)) points to another plausible mechanism through which the gut microbiome could directly influence neuroinflammation in SAE, possibly by facilitating bacterial translocation or modulating the host’s immune response to systemic infection (46).

The translational power of our findings is most evident in the biomarker discovery phase of our investigation. The convergence of two distinct and robust machine learning algorithms (LASSO and elastic net regression) on miR-30e-3p and miR-223-5p from an initial pool of 25 candidate features is a strong indicator of their biological and diagnostic significance. Their combined ability to distinguish SAE from uncomplicated sepsis with an AUC of 0.893 presents a compelling case for developing a rapid, non-invasive, stool-based diagnostic test, addressing a critical unmet need in the ICU where SAE is currently a diagnosis of exclusion based on clinical assessment alone. Such a test could enable earlier identification of high-risk patients, facilitate timely intervention, and improve patient stratification in future clinical trials, ultimately addressing the critical need for specific biomarkers in SAE management (47, 48).

Furthermore, our integrated functional analysis using ceRNA network construction and subsequent experimental validation provides a plausible mechanistic framework for the role of these miRNAs in SAE pathogenesis. The predicted targeting of IL-1β by miR-30e-3p is highly consequential given the established role of IL-1β as a master regulator of neuroinflammation in SAE (49). Our prior work has demonstrated that IL-1β promotes hippocampal neuron damage in an autophagy-dependent manner, and sustained elevation of this cytokine has been closely associated with neuronal loss and cognitive deficits in experimental models of sepsis (7). The significant elevation of serum IL-1β in our SAE patient cohort provides crucial translational evidence that this pathway is actively involved in human SAE and suggests that miR-30e-3p may be a key upstream regulator of this inflammatory cascade (50). Concurrently, miR-223-5p’s predicted targeting of immune-regulatory genes (*IGF1*, *AR*) aligns with its established role in promoting M1 microglial activation, proinflammatory cytokine expression, and neuronal apoptosis through downregulation of neuroprotective neuregulin-1 and activation of caspase-3 (48, 51). The simultaneous dysregulation of both miRNAs suggests a potentially synergistic effect, driving a feed-forward loop of inflammation and immune dysfunction that characterizes the complex pathophysiology of SAE. This multi-target action may explain their superior diagnostic performance when combined as a panel.

From a mechanistic standpoint, the precise mode of interaction within the gut-microbiota-fecal miRNA network warrants deeper investigation. In addition to microbiota colonization models, future work should also assess neutrophil activation and NETosis in response to gut-derived miRNA or microbial product translocation, to directly test whether gut-miRNA changes can trigger immune-mediated brain injury as described in recent sepsis studies (37). While our study and others demonstrate compelling correlations (52), establishing causality requires functional validation. Future research should employ gnotobiotic animal models colonized with specific bacterial taxa identified here (e.g., *Neisseria*, *Haemophilus*) to determine their direct impact on host miR-30e-3p and miR-223-5p expression in the gut and the brain. Conversely, administering miRNA agonists or antagonists could elucidate their effect on the gut microbial composition, potentially closing the loop on this bidirectional communication.

## Limitations

Despite these strengths, several limitations must be acknowledged and addressed in future research. The sample size, though sufficient for this initial discovery-phase study, warrants validation in a larger, multi-center cohort to ensure generalizability and strengthen statistical power for subgroup analyses. The correlative nature of the microbiota-miRNA interactions, while hypothesis-generating, necessitates future functional studies using gnotobiotic animal models, *in vitro* co-culture systems, or targeted miRNA modulation experiments to establish causality and directionality in these relationships. The absence of paired brain tissue samples limits our ability to definitively conclude that the observed fecal miRNA changes directly reflect or directly influence processes within the CNS, though their strong correlation with clinical markers of neurological dysfunction supports their utility as peripheral biomarkers of central processes. Additionally, while we controlled for major confounders through careful patient matching, the potential impact of specific antibiotics, sedatives, and other ICU medications on the microbiome-miRNA axis could be explored in future studies with detailed pharmacovigilance data. Finally, the relatively high prevalence of intestinal source infections in both groups, while not statistically different, warrants further investigation in a larger cohort to determine whether gut-origin sepsis has a distinct effect on the microbiome-miRNA-brain axis compared to other infection sources.

## 5 Conclusion

This study fundamentally advances the understanding of SAE by illuminating the gut-microbiota-fecal miRNA axis as a novel and critical pathway in its pathogenesis. We provide not only a mechanistic model of gut-brain communication but also a translatable diagnostic solution with immediate clinical potential. The miRNA signature we identified offers a promising, non-invasive tool for early SAE detection, which could facilitate timely intervention and improve patient stratification in future clinical trials. Beyond diagnosis, our findings open new therapeutic avenues aimed at modulating the gut microbiome or targeting specific miRNA pathways to mitigate neuroinflammation and improve neurological outcomes in septic patients. Future research should focus on validating these biomarkers in larger cohorts, elucidating the precise mechanistic pathways through which these miRNAs influence neuroinflammation, and exploring targeted interventions that could disrupt this deleterious gut-brain communication in sepsis.

## Data Availability

The datasets presented in this study can be found in online repositories. The names of the repository/repositories and accession number(s) can be found below: https://www.ncbi.nlm.nih.gov/, PRJNA1134079; https://www.ncbi.nlm.nih.gov/, PRJNA1134261.
