# Supplementary material for: Intestinal microenvironment dynamics and Sepsis-associated encephalopathy pathophysiology: insights from multi-omics profiling
Source: Front Neurol. 2026 Jan 26;16:1724644. doi: 10.3389/fneur.2025.1724644 (PMC12884059; doi:10.3389/fneur.2025.1724644)
Supplement: Supplementary file 1 [file Data_Sheet_1.pdf]

Text S1. The inclusion and exclusion criteria

Inclusion Criteria

1. Sepsis (SP) patients: Patients meeting the diagnostic criteria for sepsis based on the "Sepsis 3.0" guidelines established at the 2016 International Conference on Definitions of Sepsis by the Society of Critical Care Medicine (SCCM), the European Society of Intensive Care Medicine (ESICM), the American College of Chest Physicians (ACCP), the American Thoracic Society (ATS), and the Surgical Infection Society (SIS).

2. Sepsis-associated encephalopathy (SAE) patients: Patients diagnosed with sepsis who exhibit documented cognitive and neuropsychiatric impairments recorded by clinicians and nurses, with a Glasgow Coma Scale (GCS) score <15 and delirium confirmed by the Confusion Assessment Method for the ICU (CAM-ICU). Manifestations of delirium include inattention, disorientation, altered thinking, psychomotor slowing, or agitation. In addition, SAE-specific electroencephalogram (EEG) abnormalities detected via amplitude-integrated EEG (αEEG) multimodal brain function monitoring were required, including: (1) Excessive θ-wave activity (low voltage, frequency generally >4 Hz but <8 Hz). (2) δ-wave dominance (medium to high voltage, frequency ≤4 Hz). (3) Triphasic waves (typical or atypical triphasic patterns as the main abnormality). (4) Suppression pattern (active electrode voltage <20 μV in bipolar or referential montage, indicating cerebral quiescence) or burst-suppression pattern.

Exclusion Criteria

1. Primary neurological diseases, including central nervous system infections (e.g.,

meningitis, encephalitis caused by various pathogens), cerebrovascular diseases (e.g., cerebral hemorrhage, cerebral infarction), autoimmune encephalitis, or epilepsy.

2. non-neurological diseases, including metabolic disorders (e.g., electrolyte imbalance, hypoglycemia, diabetic ketoacidosis, hepatic encephalopathy, pulmonary encephalopathy, or uremic encephalopathy).

3. Rheumatic and hematological disorders (e.g., hemolytic-uremic syndrome, systemic lupus erythematosus).

4. Toxicity and withdrawal symptoms (e.g., alcohol or carbon monoxide poisoning).

5. Cognitive and consciousness changes related to sedative or analgesic use.

Text S2. Other materials and methods

miRNA sequencing

miRNA sequencing was performed on the Illumina HiSeq 2500 platform using single-end 50 bp (SE50) reads. Small RNA libraries were prepared through adapter ligation, reverse transcription with SuperScript II, PCR amplification with indexed primers, and PAGE gel purification to select target fragments (~147-157 nt including adapters). Library quality was assessed using an Agilent 2100 Bioanalyzer, and sequencing-ready libraries were denatured with NaOH and stored at -20°C in 10 mM Tris-HCl (pH 8.5) with 0.1% Tween-20. After sequencing, clean reads were aligned to the miRBase v22 mature miRNA database, and only sequences annotated as hsa- (Homo sapiens) were retained. Reads not matching hsa-miRNAs, as well as those mapping to rRNA or tRNA, were excluded. Length filtering (18-30 nt) was also applied to ensure specificity of miRNA detection.

16S rDNA sequencing

16S rDNA sequencing was conducted using the Illumina NovaSeq 6000 platform with paired-end 250 bp (PE250) reads via a NovaSeq 6000 SP reagent kit (500 cycles). Total microbial DNA was extracted via the CTAB method, and the V3-V4 region was PCR-amplified using specific primers. The PCR products were purified using AMPure XT beads and quantified via an Agilent 2100 Bioanalyzer and an Illumina KAPA library quantification kit. The final libraries were diluted, denatured, and sequenced according to standard Illumina protocols.

Enzyme-linked immunosorbent assay (ELISA)

Serum IL-1β levels were quantified using a human IL-1β ELISA kit (Elabscience®, Wuhan, China) via a double-antibody sandwich method. Samples and standards were added to microplates precoated with capture antibodies, followed by incubation with

biotinylated detection antibodies and horseradish peroxidase (HRP)-conjugated streptavidin. After washing, TMB substrate was added, and the absorbance was measured at 450 nm. Concentrations were calculated from a standard curve. All the samples were processed according to the manufacturer’s instructions, centrifuged at 2-8 °C, and screened for hemolysis or lipemia. Assays were performed in duplicate with appropriate controls. The detection range was 7.81–500 pg/mL, the sensitivity was 4.69 pg/mL, and the intra/-/interassay coefficients of variability (CVs) were <10%.

Co-Occurrence Network Construction

After obtaining the correlation coefficient matrix between genera and miRNAs, a series of stringent filtering criteria were applied: (a) weak correlations (coefficients ≤ 0.6) were excluded; (b) self-loops were removed to ensure meaningful connections; and (c) connections involving nodes with relative abundances below 0.005% were discarded. The filtered correlation matrix was then used to construct a co-occurrence network in Cytoscape (Version 3.9.1), where nodes represented bacterial genera or miRNAs, and edges represented significant correlations. This network provides a visual framework for understanding the intricate interactions between bacterial genera and miRNAs, highlighting key relationships with potential biological relevance.

Lasso Regression Algorithm and Elastic Net Regression Algorithm

We incorporated 25 significantly correlated miRNAs and microorganisms into analyses using the Lasso regression algorithm and the elastic net regression algorithm, both implemented via the R package glmnet. The Lasso regression algorithm was employed to eliminate collinear variables, utilizing default parameter settings. For the elastic net

regression analysis, the alpha parameter was set to 0.8, and the regularization parameter λ was selected based on the lambda.1se criterion, ensuring optimal model performance.

Supplementary Tables

Table S1. Comparison of baseline characteristics of patients

Variables	Total (n = 30)	SP (n = 20)	SAE (n = 10)	p
**Age（years）**	75.0(64.3– 85.8)	71.0(64.0– 82.7)	77.0(74.5– 87.5)	0.201
**Gender, n (%)**				0.425
Man	11 (37)	6 (30)	5 (50)	
Woman	19 (63)	14 (70)	5 (50)	
**Underlying diseases, n (%)**				
DM	12 (40)	8 (40)	4 (40)	>0.999
Hypertension	16 (53)	10 (50)	6 (60)	0.709
CVD	4 (13)	3 (15)	1 (10)	>0.999
COPD	5 (17)	3 (15)	2 (20)	>0.999
CHD	12 (40)	6 (30)	6 (60)	0.139
CKD	8 (27)	7 (35)	1 (10)	0.21
Cirrhosis	3 (10)	2 (10)	1 (10)	>0.999
Tumor	4 (13)	3 (15)	1 (10)	>0.999
**Disease severity**				
SOFA Score	7.0(4.3–10.8)	6.0(4.8–10.6)	8.0(4.5–11.0)	0.58
APACHE II Score				
Admission	18.0(12.5–22.8)	18.0(15.8– 23.3)	15.5(11.3–20.3)	0.233
72 hours	7.0(5.25-11.0)	7.0(5.0-11.0)	7.0(6.0-10.25)	0.526
GCS, n (%)				0.001**
3	1 (3.3)	0 (0)	1 (10)	
4	1 (3.3)	0 (0)	1 (10)	
9	1 (3.3)	0 (0)	1 (10)	
12	4 (13)	1 (5.0)	3 (30)	
13	5 (17)	2 (10)	3 (30)	
14	1 (3.3)	0 (0)	1 (10)	
15	17 (57)	17 (85)	0 (0)	
**Outcome, n (%)**				
28-day survival	21 (70)	14 (70)	7 (70)	>0.999
90-day survival	19 (63)	13 (65)	6 (60)	>0.999

Note: SP, Sepsis; SAE, Sepsis-associated encephalopathy; DM, Diabetes Mellitus; CVD, Cardiovascular Disease; COPD, Chronic Obstructive Pulmonary Disease; CHD, Coronary Heart Disease; CKD, Chronic Kidney Disease; SOFA, sequential organ failure assessment; APACHE II, acute physiology, age and chronic health evaluation II. GCS, Glasgow Coma Scale. **$p < 0.01$.

Table S2. Comparison of clinical and biochemical indexes in patients

Variables	Total (n = 30)	SP (n = 20)	SAE (n = 10)	*p*
**Site of infection, n (%)**				0.950
Biliary tract	3(10)	2(10)	1(10)	
Intestinal tract	12(40)	7(35)	5(50)	
Lung	12(40)	8(40)	4(40)	
Skin	1(3.3)	1(5.0)	0(0)	
Urinary tract	2(6.7)	2(10)	0(0)	
**Biochemical Indexes (median)**				
TNF-α (pg/L)	0.97(0.67–2.13)	0.85(0.73–2.13)	1.38(0.64–2.57)	0.660
IL-2 (pg/L)	1.65(0.77–2.32)	1.44(1.06–2.22)	1.77(0.73–2.29)	0.840
IL-4 (pg/L)	1.07(0.76–1.57)	1.04(0.79–1.44)	1.07(0.75–1.75)	0.470
IL-6 (pg/L)	342.72(214.77–911.73)	277.53(216.38–722.27)	486.27(192.42–1118.12)	0.340
IL-10 (pg/L)	16.84(5.44–46.84)	9.88(5.87–16.84)	22.62(5.44–49.20)	0.300
IFN-γ (pg/L)	0.99(0.34–1.54)	0.99(0.34–1.38)	1.08(0.22–1.84)	0.890
WBC ($\times 10^9$ /L)	12.20(6.85–18.13)	11.40(9.60–22.05)	13.10(6.13–17.38)	0.450
HCT	0.32(0.27–0.38)	0.36(0.31–0.40)	0.27(0.25–0.30)	0.009^{**}
PLT ($\times 10^9$ /L)	145.50(83.50–177.75)	147.50(123.75–208.25)	123.50(62.75–154.00)	0.150
CRP（mg/dL）	138.55(79.30–238.48)	138.55(77.45–254.88)	166.00(92.23–222.08)	0.750
TBil（umol/L）	19.55(9.80–29.45)	19.55(9.58–25.28)	21.75(10.43–38.08)	0.760
Scr（umol/L）	143.50(113.75–162.50)	149.50(114.25–205.25)	134.00(115.00–144.00)	0.130
PO_2/FiO_2（mmHg）	275.05(232.50–330.25)	296.70(262.65–383.41)	243.65(207.08–263.92)	0.044^{*}
LAC（mmol/ml）	2.31(1.52–2.94)	1.55(1.37–2.32)	2.49(2.18–5.62)	0.048^{*}
MAP（mmHg）	78.50(71.75–83.50)	78.34(71.92–82.67)	79.50(71.75–86.00)	0.910

Note: SP, Sepsis; SAE, Sepsis-associated encephalopathy; TNF-α, Tumor Necrosis Factor-alpha; IL-2, Interleukin 2; IL-4, Interleukin 4; IL-6, Interleukin 6; IL-10, Interleukin 10; IFN-γ, Interferon γ; WBC, White blood cell; HCT, Hematocrit; PLT, Platelet; CRP, C-Reactive Protein; TBil, Total Bilirubin; Scr, Serum Creatinine; PO_2: Oxygen Permeance; FiO_2: Fraction of inspiration oxygen; LAC, Lactate; MAP, Mean Arterial Pressure. $^{*}p < 0.05$, $^{**}p < 0.01$.

Table S3. Expression of 12 differentially expressed miRNAs in SAE

Mature_ID	baseMean	log2FoldChange	p-value	type
hsa-miR-106a-5p	4.72022805	2.837255257	0.001796672	Up
hsa-miR-181a-5p	245.4162488	1.250836611	0.000280138	Up
hsa-miR-222-3p	104.0215838	-1.757563717	0.000813901	Down
hsa-miR-223-5p	7.148572148	3.221989473	4.75E-05	Up
hsa-miR-223-3p	565.6993958	3.76188861	1.57E-06	Up
hsa-miR-140-3p	22.45942157	1.656652672	0.000131402	Up
hsa-miR-30e-3p	23.54543225	1.320662772	0.000604408	Up
hsa-miR-363-3p	43.193939	2.504354183	0.000174567	Up
hsa-miR-378a-3p	4630.625216	2.158268354	0.000726568	Up
hsa-miR-542-3p	2.983306669	3.109623298	0.001805185	Up
hsa-miR-769-5p	25.89121229	2.651317097	3.64E-05	Up
hsa-miR-378d	8.918849318	2.706047731	0.000486703	Up

Table S4 The correlation between DE miRNAs and SAE-associated bacterial

Taxonomy	r-value	p-value	FDR corrected p-value
hsa-miR-106a-5p			
Prevotella	-0.362	0.050	0.037
Flavonifractor	-0.411	0.024	0.031
Neisseria	0.393	0.032	0.034
Gardnerella	-0.487	0.006	0.042
Ureaplasma	-0.388	0.034	0.017
Elizabethkingia	-0.522	0.003	0.008
hsa-miR-181a-5p			
Bacteroides	-0.465	0.010	0.011
Prevotella	-0.364	0.048	0.021
hsa-miR-222-3p			
Oscillibacter	-0.594	0.001	0.026
Massilimicrobiota	-0.472	0.008	0.017
Neisseria	-0.421	0.021	0.029
hsa-miR-223-5p			
Bacteroides	-0.514	0.004	0.019
Phocaeicola	-0.518	0.003	0.031
Ureaplasma	-0.365	0.047	0.044
Elizabethkingia	-0.402	0.028	0.028
hsa-miR-140-3p			
Methanobrevibacter	0.413	0.023	0.021
Massilimicrobiota	0.376	0.040	0.027
Neisseria	0.399	0.029	0.033
Haemophilus	0.573	0.001	0.009
Ureaplasma	-0.508	0.004	0.019
Elizabethkingia	-0.420	0.021	0.046
Lautropia	0.372	0.043	0.022
hsa-miR-30e-3p			
Granulicatella	-0.435	0.016	0.020
Oscillibacter	0.504	0.004	0.018
Massilimicrobiota	0.466	0.009	0.026
hsa-miR-363-3p			
Flavonifractor	-0.418	0.022	0.027
Ureaplasma	-0.439	0.015	0.030
Elizabethkingia	-0.501	0.005	0.021
hsa-miR-378a-3p			
Lactococcus	-0.361	0.050	0.017
hsa-miR-542-3p			
Methanobrevibacter	0.392	0.032	0.010
Oscillibacter	0.421	0.020	0.016
Neisseria	0.362	0.0490	0.0230
Ureaplasma	-0.421-	.0200.	.0170.
Elizabethkingia	0.372	043	035
hsa-miR-769-5p			
Elizabethkingia	-0.362	0.049	0.024

Table S5. Clinical Associations of SAE-Related miRNAs and Microbiota

Data1	Data2	rho	*p*-value	relation
GCS	Ureaplasma	-0.508539715	0.004111364	negative
IL-2	Neisseria	0.526056047	0.002827955	positive
LAC	miR-106a-5p	0.525171719	0.002883259	positive
LAC	miR-140-3p	0.549027614	0.001678288	positive
